# Supplementary material for: In Silico Evaluation of Bioactive Compounds of Artemisia pallens Targeting the Efflux Protein of Multidrug-Resistant Acinetobacter baumannii (LAC-4 Strain)
Source: Molecules. 2022 Aug 15;27(16):5188. doi: 10.3390/molecules27165188 (PMC9414700; doi:10.3390/molecules27165188)
Supplement: Supplementary file 1 [file molecules-27-05188-s001.zip › molecules-1781676-supplementary.pdf]

Supplementary Table

Table S1: List of extricated compounds attained from GC–MS Assay

Sl. no	Retention time	Name of the compound	Peak area (%)
1	RT-10.164	Lilac alcohol A	7.29
2	RT-11.108	3,5-Dimethylanisole	1.81
3	RT-11.797	Davana ether	2.79
4	RT-12.564	5-Hepten-3-one, 2-(5-ethenyltetrahydro-5-methyl-2-furanyl)-6-methyl-, [2S-[2.alpha.(R*),5.alpha.]]-	2.76
5	RT-12.652	1H-Cycloprop[e]azulen-7-ol, decahydro-1,1,7-trimethyl-4-methylene-, [1ar-(1a.alpha.,4a.alpha.,7.beta., 7a.beta.,7b.alpha.)]-	3.03
6	RT-13.163	5,7-Octadien-3-ol, 2,4,4,7-tetramethyl-, (E)-	1.08
7	RT-13.275	1-Cyclopentene-1-methanol, 2-methyl-5-(1-methylethyl)-	1.36
8	RT-13.619	Silane, trimethyl(methylenecyclopropyl)-	1.41
9	RT-13.663	7-Oxabicyclo[2.2.1]heptane, 1-methyl-4-(1-methylethyl)-	0.97
10	RT-13.863	Lilac alcohol C	32.77
11	RT-14.086	4-((1E)-3-Hydroxy-1-propenyl)-2-methoxyphenol	1.38
12	RT-15.752	cis-Z-.alpha.-Bisabolene epoxide	0.96
13	RT-15.974	n-Hexadecanoic acid	7.92
14	RT-17.163	Phytol	1.67
15	RT-17.341	9,12-Octadecadienoic acid (Z,Z)-	2.94
16	RT-17.430	Naphtho[1,2-b]furan-2,6(3H,4H)-dione, 3a,5,5a,9,9a,9b-hexahydro-9-hydroxy-3,5a,9-trimethyl-	15.14
17	RT-17.496	Benzoic acid, 3,5-bis(1,1-dimethylethyl)-4-hydroxy-, methyl ester	1.65
18	RT-17.541	16-Methyloxacyclohexadeca-3,5-dien-2-one	1.90
19	RT-18.274	Octacosyl acetate	1.97
20	RT-18.641	Ethyl 4-acetylbenzoate	1.03
21	RT-19.007	Cyclooctaneacetic acid, 2-oxo-	1.37
22	RT-20.007	Hexadecanoic acid, 2-hydroxy-1-(hydroxymethyl)ethyl ester	1.73
23	RT-21.174	1-Nonadecene	2.67
24	RT-22.340	Adamantane-1,3-diol	0.89
25	RT-22.396	Heptadecane	1.51

Supplementary Figure

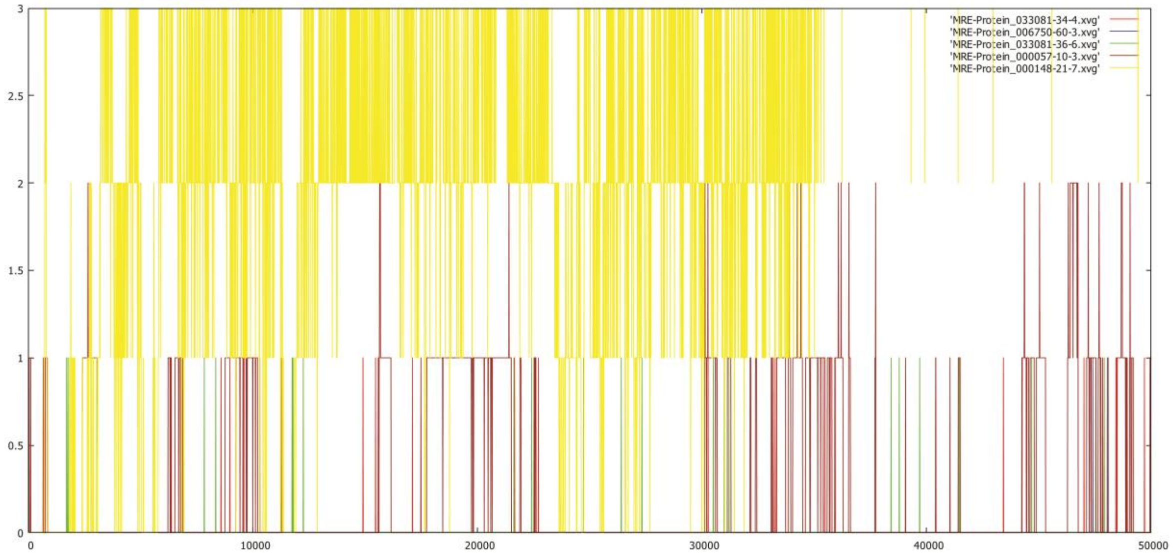

Figure S1: Total number of inter-molecular hydrogen bond (H_bond) interactions between the ligands (033081-34-4, 006750-60-3, 033081-36-6, 000057-10-3 and 000148-21-7 efflux protein of *A. baumannii* (LAC-4))
